# Crystal structure of 1-{4-hy­droxy-3-[(pyrrolidin-1-yl)meth­yl]phen­yl}-3-phenyl­prop-2-en-1-one

**DOI:** 10.1107/S2056989016006009

**Published:** 2016-04-15

**Authors:** Abdullah Aydın, Mehmet Akkurt, Halise Inci Gul, Kadir Ozden Yerdelen, Raziye Catak Celik

**Affiliations:** a Department of Science Education, Faculty of Education, Kastamonu University, 37200 Kastamonu, Turkey; b Department of Physics, Faculty of Sciences, Erciyes University, 38039 Kayseri, Turkey; c Department of Pharmaceutical Chemistry, Faculty of Pharmacy, Atatürk University, 25240 Erzurum, Turkey; d Scientific and Technological Application and Research Center, Aksaray University, 68100 Aksaray, Turkey

**Keywords:** crystal structure, Mannich bases, semi-empirical, methyl­phen­yl, intra­molecular O—H⋯N hydrogen bond

## Abstract

In the title compound, C_20_H_21_NO_2_, the pyrrolidine ring adopts an envelope conformation with the N atom at the flap position. The central benzene ring makes dihedral angles of 21.39 (10) and 80.10 (15)° with the phenyl ring and the mean plane of the pyrrolidine ring, respectively. The mol­ecular conformation is stabilized by an intra­molecular O—H⋯N hydrogen bond, which closes an *S*(6) ring. A weak C—H⋯π inter­action is observed in the crystal.

## Abstract 2

In the title compound, the pyrrolidine ring adopts an envelope conformation, which may be correlated with the intra­molecular O—H⋯N hydrogen bond.

## Chemical context   

Mannich bases are a group of compounds having various biological activities such as cytotoxic (Bilginer *et al.*, 2013 ▸), anti-inflammatory (Sahin *et al.*, 2010 ▸) and anti­convulsant (Gul *et al.*, 2004 ▸) activities. α,β-Unsaturated ketones present in the chemical structure of Mannich bases themselves or those produced from them by deamination processes are responsible for their cytotoxicity.
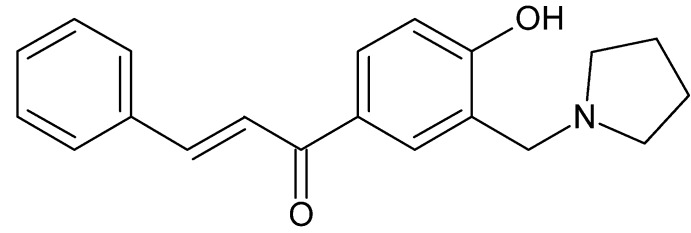


The cytotoxic and anti­cancer properties of chalcone (1,3-diphenyl-2-propenone) and related compounds have been reported (Bilginer *et al.*, 2013 ▸; Dimmock *et al.*, 1998 ▸; Gul Cizmecioglu *et al.*, 2009 ▸); Gul Mete *et al.*, 2009 ▸). The title compound, (I), reported in this study is a Mannich base of phenolic chalcone.

## Structural commentary   

**Figure 1:**
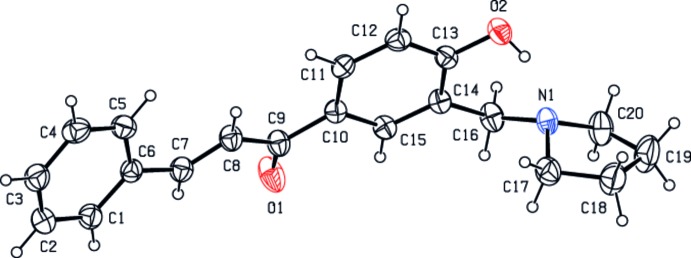
View of the mol­ecular structure of the title compound, with displacement ellipsoids drawn at the 30% probability level.

**Figure 2:**
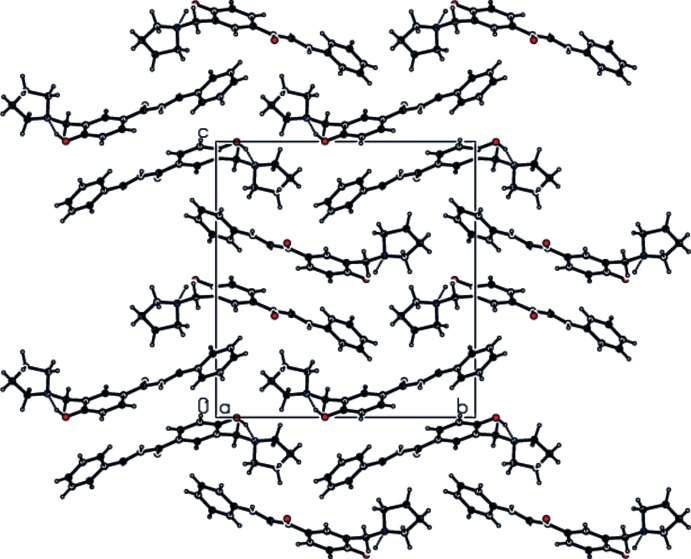
The mol­ecular packing and hydrogen bonding viewed down the *a* axis.

In the title compound (Fig. 1 ▸), the pyrrolidine ring (N1/C17–C20) exhibits an envelope conformation with the N atom at the flap position [the puckering parameters are *Q*(2) = 0.350 (3) Å and φ(2) = 186.9 (5)°]. The central benzene ring (C10–C15) makes dihedral angles of 21.39 (10) and 80.10 (15)°, with the phenyl ring (C1–C6) and the mean plane of the pyrrolidine ring (N1/C17–C20), respectively. Otherwise, the geometrical parameters for (I) are comparable those reported for related compounds (Suhud *et al.*, 2015 ▸; Palakshamurthy *et al.*, 2012 ▸). An intra­molecular O2—H1*O*⋯N1 hydrogen bond (Table 1 ▸, Fig. 2 ▸) helps to establish the mol­ecular conformation of (I).

## Supra­molecular features   

The only directional inter­action present in the crystal of (I) is a very weak C—H⋯π bond (Table 1 ▸).

## Semi-empirical quantum-mechanical calculations   

**Figure 3:**
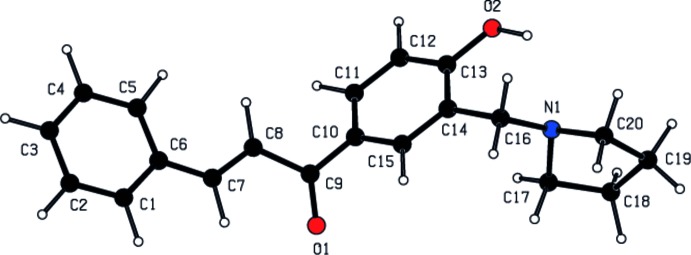
The conformation of the title compound, calculated using the *CNDO* method.

A theoretical calculation was carried out using the semi-empirical quantum-mechanical *CNDO/2* (Complete Neglect of Differential Overlap) method (Pople & Beveridge, 1970 ▸). The spatial view of the single mol­ecule, with atomic labels, calculated as a closed-shell in a vacuum is shown in Fig. 3 ▸. The charges at atoms O1, O2 and N1 are −0.337, −0.271 and −0.159 e^−^, respectively. The calculated dipole moment is 2.760 Debye.

## Biological activity   

Compound (I) was tested against human hepatoma (Huh7) and breast cancer cell (T47D) lines in terms of its cytotoxic activities, and showed activities against both cell lines used, especially against the T47D cell line. The compound studied here may serve as a model compound for designing new anti­cancer compounds for further studies (Yerdelen, 2009 ▸).

## Synthesis and crystallization   

A solution of paraformaldehyde (0.132 g; 4.4 mmol) and pyrrolidine (0.317 g, 4.4 mmol) in aceto­nitrile (5 mL) was heated under reflux at 353 K for 30 min. A solution of the chalcone, 1-(4-hy­droxy­phen­yl)-3-phenyl-2-propen-1-one (1 g, 4.4 mmol) in aceto­nitrile (25 ml), was added to the reaction flask and heating was continued. The reaction was monitored by thin layer chromatography (TLC) and stopped after 7.5 h. The reaction solvent was distilled under vacuum. The residue was purified by column chromatography using Al_2_O_3_ as adsorbant and CHCl_3_/MeOH (9:1) as eluent. The title compound was obtained in 44% yield (m.p. = 398–402 K).Crystals suitable for X-ray diffaction analysis were obtained by recrystallization from ehanol.

^1^H NMR (CDCl_3_, p.p.m.) δ 1.89–1.86 (*m*, 4H, C18-H, C19-H); 2.67 (*br s*, 4H, C17-H, C20-H); 3.90 (*s*, 2H, C16-H); 6.88–6.86 (*d*, 1H, C14-H); 7.41–7.39 (*m*, 3H, C3-H, C4-H, C5-H); 7.56–7.53 (*d*, 1H, C8-H, *J* = 15.4 Hz); 7.65–7.62 (*m*, 2H, C2-H, C6-H); 7.78–7.77 (*d*, 1H, C11-H); 7.80–7.76 (*d*, 1H, C7-H, *J* = 15.4 Hz); 7.92–7.90 (*dd*, 1H, C15-H);

^13^C NMR (CDCl_3_, p.p.m.) δ 188.82 (C9), 163.59 (C13), 143.77 (C7), 135.42 (C1), 130.43 (C11), 130.39 (C15), 129.60 (C10), 129.25 (C3, C5), 129.12 (C4), 128.55 (C2, C6), 122.68 (C12), 122.16 (C8), 116.15 (C14), 50.80 (C16), 53.69 (C17, C20), 23.88 (C18, C19); TOF MS [ES (−)] (CHCl_3_) *m*/*z*: *M* ^+^ (307.15), *M* ^+^-1 (306.15) (Yerdelen, 2009 ▸).

## Refinement   

Crystal data, data collection and structure refinement details are summarized in Table 2 ▸. Carbon-bound H atoms were placed in calculated positions with C—H = 0.93 and 0.97 Å, and refined using a riding model with *U*_iso_(H) = 1.2*U*_eq_(C). The hydroxyl H atom was found from a difference Fourier map and its positional parameters were freely refined with *U*_iso_(H) = 1.5*U*_eq_(O). The most disagreeable reflections (2 4 0), (4 9 0), (4 12 0), (5 12 4), (3 12 5), (3 3 1), (0 16 5), (1 3 0), (2 20 6), (−2 13 17), (0 5 4), (0 11 4) and (2 13 4) were omitted in the final cycles of refinement. The Flack absolute structure parameter was found to be indeterminate in the present study.

## Supplementary Material

Crystal structure: contains datablock(s) global, I. DOI: 10.1107/S2056989016006009/hb7576sup1.cif

Structure factors: contains datablock(s) I. DOI: 10.1107/S2056989016006009/hb7576Isup2.hkl

Supporting information file. DOI: 10.1107/S2056989016006009/hb7576Isup3.cml

CCDC reference: 1473395

Additional supporting information:  crystallographic information; 3D view; checkCIF report

## Figures and Tables

**Table 1:** Hydrogen-bond geometry (Å, °) *Cg*3 is the centroid of the C10–C15 ring.

*D*—H⋯*A*	*D*—H	H⋯*A*	*D*⋯*A*	*D*—H⋯*A*
O2—H1*O*⋯N1	0.85 (3)	1.85 (3)	2.633 (2)	154 (3)
C5—H5⋯*Cg*3^i^	0.93	2.99	3.685 (2)	132

Symmetry code: (i) 
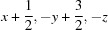
.

**Table 2:** Experimental details

Crystal data	
Chemical formula	C_20_H_21_NO_2_
*M*_r_	307.38
Crystal system, space group	Orthorhombic, *P*2_1_2_1_2_1_
Temperature (K)	296
*a*, *b*, *c* (Å)	5.8403 (5), 16.3195 (13), 17.3615 (14)
*V* (Å^3^)	1654.7 (2)
*Z*	4
Radiation type	Mo *K*α
μ (mm^−1^)	0.08
Crystal size (mm)	0.66 × 0.53 × 0.33

Data collection	
Diffractometer	Bruker APEXII CCD
Absorption correction	Multi-scan (*SADABS*; Bruker, 2007 ▸)
*T*_min_, *T*_max_	0.951, 0.974
No. of measured, independent and observed [*I* > 2σ(*I*)] reflections	37526, 4120, 3647
*R*_int_	0.050
(sin θ/λ)_max_ (Å^−1^)	0.668

Refinement	
*R*[*F* ^2^ > 2σ(*F* ^2^)], *wR*(*F* ^2^), *S*	0.043, 0.118, 1.03
No. of reflections	4120
No. of parameters	211
No. of restraints	1
H-atom treatment	H atoms treated by a mixture of independent and constrained refinement
Δρ_max_, Δρ_min_ (e Å^−3^)	0.24, −0.12

Computer programs: *APEX2* and *SAINT* (Bruker, 2007 ▸), *SHELXS97* and *SHELXL97* (Sheldrick, 2008 ▸), *ORTEP-3 for Windows* (Farrugia, 2012 ▸) and *PLATON* (Spek, 2009 ▸).

## supplementary crystallographic information

### Crystal data

**Table:**

C_20_H_21_NO_2_	*F*(000) = 656
*M**_r_* = 307.38	*D*_x_ = 1.234 Mg m^−^^3^
Orthorhombic, *P*2_1_2_1_2_1_	Mo *K*α radiation, λ = 0.71073 Å
Hall symbol: P 2ac 2ab	Cell parameters from 4120 reflections
*a* = 5.8403 (5) Å	θ = 2.4–27.7°
*b* = 16.3195 (13) Å	µ = 0.08 mm^−^^1^
*c* = 17.3615 (14) Å	*T* = 296 K
*V* = 1654.7 (2) Å^3^	Prism, light yellow
*Z* = 4	0.66 × 0.53 × 0.33 mm

### Data collection

**Table:**

Bruker APEXII CCD diffractometer	4120 independent reflections
Radiation source: sealed tube	3647 reflections with *I* > 2σ(*I*)
Graphite monochromator	*R*_int_ = 0.050
φ and ω scans	θ_max_ = 28.4°, θ_min_ = 1.7°
Absorption correction: multi-scan (*SADABS*; Bruker, 2007)	*h* = −7→7
*T*_min_ = 0.951, *T*_max_ = 0.974	*k* = −21→21
37526 measured reflections	*l* = −23→23

### Refinement

**Table:**

Refinement on *F*^2^	1 restraint
Least-squares matrix: full	Hydrogen site location: mixed
*R*[*F*^2^ > 2σ(*F*^2^)] = 0.043	H atoms treated by a mixture of independent and constrained refinement
*wR*(*F*^2^) = 0.118	*w* = 1/[σ^2^(*F*_o_^2^) + (0.068*P*)^2^ + 0.1361*P*] where *P* = (*F*_o_^2^ + 2*F*_c_^2^)/3
*S* = 1.03	(Δ/σ)_max_ < 0.001
4120 reflections	Δρ_max_ = 0.24 e Å^−^^3^
211 parameters	Δρ_min_ = −0.12 e Å^−^^3^

### Special details

**Table:**

Geometry. Bond distances, angles etc. have been calculated using the rounded fractional coordinates. All su's are estimated from the variances of the (full) variance-covariance matrix. The cell esds are taken into account in the estimation of distances, angles and torsion angles
Refinement. Refinement on F^2^ for ALL reflections except those flagged by the user for potential systematic errors. Weighted R-factors wR and all goodnesses of fit S are based on F^2^, conventional R-factors R are based on F, with F set to zero for negative F^2^. The observed criterion of F^2^ > 2sigma(F^2^) is used only for calculating -R-factor-obs etc. and is not relevant to the choice of reflections for refinement. R-factors based on F^2^ are statistically about twice as large as those based on F, and R-factors based on ALL data will be even larger.

### Fractional atomic coordinates and isotropic or equivalent isotropic displacement parameters (Å^2^)

**Table:**

	*x*	*y*	*z*	*U*_iso_*/*U*_eq_	
O1	−0.0436 (3)	0.72582 (11)	0.13185 (14)	0.0800 (7)	
O2	0.4920 (3)	0.42033 (9)	0.00124 (10)	0.0578 (5)	
N1	0.1759 (3)	0.34948 (10)	0.08639 (10)	0.0495 (5)	
C1	0.3003 (4)	0.98751 (12)	0.23067 (13)	0.0524 (6)	
C2	0.4274 (5)	1.05592 (14)	0.25148 (13)	0.0607 (7)	
C3	0.6363 (4)	1.06897 (14)	0.21774 (14)	0.0610 (7)	
C4	0.7188 (4)	1.01504 (15)	0.16329 (13)	0.0602 (7)	
C5	0.5935 (4)	0.94596 (13)	0.14348 (12)	0.0534 (6)	
C6	0.3823 (3)	0.93109 (11)	0.17767 (10)	0.0443 (5)	
C7	0.2469 (4)	0.85825 (12)	0.15995 (12)	0.0498 (6)	
C8	0.3149 (4)	0.79067 (12)	0.12533 (12)	0.0511 (6)	
C9	0.1583 (4)	0.72064 (12)	0.11351 (13)	0.0499 (6)	
C10	0.2497 (3)	0.64318 (11)	0.08086 (10)	0.0431 (5)	
C11	0.4636 (4)	0.63705 (12)	0.04543 (11)	0.0466 (6)	
C12	0.5411 (4)	0.56234 (13)	0.01826 (11)	0.0495 (6)	
C13	0.4084 (3)	0.49271 (12)	0.02654 (11)	0.0441 (5)	
C14	0.1907 (3)	0.49728 (11)	0.06048 (11)	0.0433 (5)	
C15	0.1167 (3)	0.57238 (12)	0.08719 (11)	0.0441 (5)	
C16	0.0423 (4)	0.42179 (13)	0.06449 (14)	0.0550 (6)	
C17	0.2614 (5)	0.35266 (14)	0.16535 (14)	0.0648 (8)	
C18	0.3247 (6)	0.26561 (15)	0.18383 (17)	0.0787 (10)	
C19	0.1771 (7)	0.21350 (17)	0.1318 (2)	0.0941 (13)	
C20	0.0483 (5)	0.27222 (14)	0.08114 (18)	0.0719 (9)	
H1	0.15700	0.97930	0.25270	0.0630*	
H1O	0.418 (6)	0.3850 (16)	0.0268 (18)	0.0870*	
H2	0.37130	1.09250	0.28800	0.0730*	
H3	0.72280	1.11450	0.23160	0.0730*	
H4	0.85910	1.02500	0.13970	0.0720*	
H5	0.65110	0.90950	0.10720	0.0640*	
H7	0.09410	0.85980	0.17490	0.0600*	
H8	0.46510	0.78690	0.10790	0.0610*	
H11	0.55450	0.68350	0.04010	0.0560*	
H12	0.68320	0.55890	−0.00570	0.0590*	
H15	−0.02670	0.57600	0.11020	0.0530*	
H16A	−0.07880	0.43040	0.10190	0.0660*	
H16B	−0.02830	0.41250	0.01470	0.0660*	
H17A	0.14400	0.37250	0.20020	0.0780*	
H17B	0.39400	0.38830	0.16900	0.0780*	
H18A	0.29390	0.25340	0.23750	0.0950*	
H18B	0.48580	0.25600	0.17360	0.0950*	
H19A	0.27120	0.17710	0.10100	0.1130*	
H19B	0.07160	0.18070	0.16200	0.1130*	
H20A	0.04430	0.25260	0.02840	0.0860*	
H20B	−0.10750	0.27930	0.09930	0.0860*	

### Atomic displacement parameters (Å^2^)

**Table:**

	*U*^11^	*U*^22^	*U*^33^	*U*^12^	*U*^13^	*U*^23^
O1	0.0507 (9)	0.0596 (10)	0.1298 (18)	−0.0041 (7)	0.0166 (10)	−0.0278 (10)
O2	0.0555 (9)	0.0515 (8)	0.0663 (9)	0.0081 (7)	0.0056 (7)	−0.0088 (7)
N1	0.0488 (9)	0.0383 (8)	0.0613 (10)	−0.0074 (7)	−0.0064 (8)	−0.0087 (7)
C1	0.0520 (11)	0.0477 (10)	0.0574 (11)	−0.0010 (9)	0.0031 (10)	0.0005 (9)
C2	0.0739 (15)	0.0497 (11)	0.0586 (12)	−0.0030 (11)	0.0022 (11)	−0.0077 (9)
C3	0.0712 (15)	0.0524 (11)	0.0593 (12)	−0.0158 (11)	−0.0055 (11)	−0.0015 (10)
C4	0.0575 (13)	0.0671 (13)	0.0559 (11)	−0.0157 (11)	0.0028 (10)	0.0035 (10)
C5	0.0567 (12)	0.0548 (11)	0.0488 (10)	−0.0026 (9)	0.0032 (9)	−0.0034 (8)
C6	0.0480 (10)	0.0419 (9)	0.0431 (9)	0.0014 (8)	−0.0052 (7)	0.0033 (7)
C7	0.0475 (10)	0.0472 (10)	0.0547 (10)	−0.0015 (8)	−0.0001 (8)	0.0017 (8)
C8	0.0485 (11)	0.0450 (10)	0.0598 (11)	−0.0021 (8)	0.0000 (9)	−0.0025 (8)
C9	0.0444 (10)	0.0447 (10)	0.0607 (11)	−0.0001 (8)	−0.0013 (9)	−0.0034 (8)
C10	0.0427 (9)	0.0421 (9)	0.0446 (9)	0.0006 (8)	−0.0046 (7)	0.0001 (7)
C11	0.0442 (9)	0.0466 (10)	0.0490 (10)	−0.0057 (8)	0.0012 (8)	0.0048 (8)
C12	0.0407 (9)	0.0584 (11)	0.0493 (10)	0.0036 (9)	0.0068 (8)	0.0028 (8)
C13	0.0434 (10)	0.0459 (9)	0.0431 (8)	0.0052 (8)	−0.0032 (8)	−0.0026 (7)
C14	0.0388 (9)	0.0432 (9)	0.0478 (9)	−0.0009 (7)	−0.0065 (8)	−0.0035 (7)
C15	0.0339 (8)	0.0475 (9)	0.0510 (9)	0.0004 (7)	0.0005 (7)	−0.0029 (8)
C16	0.0424 (10)	0.0486 (10)	0.0741 (13)	−0.0049 (9)	−0.0064 (9)	−0.0110 (10)
C17	0.0794 (16)	0.0539 (12)	0.0610 (12)	−0.0079 (12)	−0.0102 (12)	−0.0087 (10)
C18	0.100 (2)	0.0572 (13)	0.0788 (16)	−0.0084 (14)	−0.0123 (17)	0.0062 (12)
C19	0.129 (3)	0.0513 (13)	0.102 (2)	−0.0247 (16)	−0.024 (2)	0.0075 (14)
C20	0.0704 (15)	0.0492 (12)	0.0960 (19)	−0.0208 (11)	−0.0125 (14)	−0.0097 (12)

### Geometric parameters (Å, º)

**Table:**

O1—C9	1.224 (3)	C17—C18	1.503 (3)
O2—C13	1.352 (2)	C18—C19	1.511 (5)
N1—C16	1.465 (3)	C19—C20	1.503 (4)
N1—C17	1.460 (3)	C1—H1	0.9300
N1—C20	1.467 (3)	C2—H2	0.9300
O2—H1O	0.85 (3)	C3—H3	0.9300
C1—C2	1.389 (3)	C4—H4	0.9300
C1—C6	1.387 (3)	C5—H5	0.9300
C2—C3	1.370 (4)	C7—H7	0.9300
C3—C4	1.379 (3)	C8—H8	0.9300
C4—C5	1.387 (3)	C11—H11	0.9300
C5—C6	1.390 (3)	C12—H12	0.9300
C6—C7	1.461 (3)	C15—H15	0.9300
C7—C8	1.317 (3)	C16—H16A	0.9700
C8—C9	1.478 (3)	C16—H16B	0.9700
C9—C10	1.485 (3)	C17—H17A	0.9700
C10—C15	1.397 (3)	C17—H17B	0.9700
C10—C11	1.396 (3)	C18—H18A	0.9700
C11—C12	1.383 (3)	C18—H18B	0.9700
C12—C13	1.383 (3)	C19—H19A	0.9700
C13—C14	1.403 (3)	C19—H19B	0.9700
C14—C15	1.380 (3)	C20—H20A	0.9700
C14—C16	1.508 (3)	C20—H20B	0.9700
			
C16—N1—C17	113.41 (17)	C3—C4—H4	120.00
C16—N1—C20	113.92 (18)	C5—C4—H4	120.00
C17—N1—C20	105.22 (19)	C4—C5—H5	120.00
C13—O2—H1O	104 (2)	C6—C5—H5	120.00
C2—C1—C6	121.5 (2)	C6—C7—H7	116.00
C1—C2—C3	119.3 (2)	C8—C7—H7	116.00
C2—C3—C4	120.4 (2)	C7—C8—H8	119.00
C3—C4—C5	120.3 (2)	C9—C8—H8	119.00
C4—C5—C6	120.27 (19)	C10—C11—H11	120.00
C1—C6—C7	119.54 (18)	C12—C11—H11	120.00
C5—C6—C7	122.18 (17)	C11—C12—H12	120.00
C1—C6—C5	118.28 (18)	C13—C12—H12	120.00
C6—C7—C8	127.9 (2)	C10—C15—H15	119.00
C7—C8—C9	121.6 (2)	C14—C15—H15	119.00
O1—C9—C8	120.43 (19)	N1—C16—H16A	109.00
O1—C9—C10	120.29 (19)	N1—C16—H16B	109.00
C8—C9—C10	119.27 (19)	C14—C16—H16A	109.00
C9—C10—C11	123.43 (17)	C14—C16—H16B	109.00
C9—C10—C15	118.32 (17)	H16A—C16—H16B	108.00
C11—C10—C15	118.24 (17)	N1—C17—H17A	111.00
C10—C11—C12	120.41 (19)	N1—C17—H17B	111.00
C11—C12—C13	120.4 (2)	C18—C17—H17A	111.00
O2—C13—C12	118.81 (17)	C18—C17—H17B	111.00
O2—C13—C14	120.68 (17)	H17A—C17—H17B	109.00
C12—C13—C14	120.51 (18)	C17—C18—H18A	111.00
C13—C14—C15	118.17 (17)	C17—C18—H18B	111.00
C13—C14—C16	119.78 (17)	C19—C18—H18A	111.00
C15—C14—C16	122.02 (17)	C19—C18—H18B	111.00
C10—C15—C14	122.29 (17)	H18A—C18—H18B	109.00
N1—C16—C14	111.35 (18)	C18—C19—H19A	111.00
N1—C17—C18	104.54 (19)	C18—C19—H19B	110.00
C17—C18—C19	105.3 (2)	C20—C19—H19A	111.00
C18—C19—C20	106.1 (2)	C20—C19—H19B	111.00
N1—C20—C19	104.9 (2)	H19A—C19—H19B	109.00
C2—C1—H1	119.00	N1—C20—H20A	111.00
C6—C1—H1	119.00	N1—C20—H20B	111.00
C1—C2—H2	120.00	C19—C20—H20A	111.00
C3—C2—H2	120.00	C19—C20—H20B	111.00
C2—C3—H3	120.00	H20A—C20—H20B	109.00
C4—C3—H3	120.00		
			
C16—N1—C17—C18	162.8 (2)	C8—C9—C10—C11	−14.0 (3)
C17—N1—C16—C14	67.5 (2)	C8—C9—C10—C15	164.78 (18)
C20—N1—C16—C14	−172.2 (2)	C15—C10—C11—C12	−0.7 (3)
C17—N1—C20—C19	−34.8 (3)	C11—C10—C15—C14	0.7 (3)
C20—N1—C17—C18	37.6 (3)	C9—C10—C11—C12	178.07 (19)
C16—N1—C20—C19	−159.6 (2)	C9—C10—C15—C14	−178.13 (18)
C6—C1—C2—C3	1.4 (3)	C10—C11—C12—C13	−0.6 (3)
C2—C1—C6—C5	−2.1 (3)	C11—C12—C13—O2	−178.20 (18)
C2—C1—C6—C7	177.4 (2)	C11—C12—C13—C14	1.9 (3)
C1—C2—C3—C4	0.5 (4)	C12—C13—C14—C15	−1.9 (3)
C2—C3—C4—C5	−1.6 (4)	C12—C13—C14—C16	176.13 (19)
C3—C4—C5—C6	0.8 (3)	O2—C13—C14—C15	178.24 (18)
C4—C5—C6—C7	−178.5 (2)	O2—C13—C14—C16	−3.7 (3)
C4—C5—C6—C1	1.0 (3)	C16—C14—C15—C10	−177.39 (19)
C5—C6—C7—C8	15.6 (3)	C13—C14—C15—C10	0.6 (3)
C1—C6—C7—C8	−163.9 (2)	C13—C14—C16—N1	42.4 (3)
C6—C7—C8—C9	178.1 (2)	C15—C14—C16—N1	−139.68 (19)
C7—C8—C9—C10	−174.3 (2)	N1—C17—C18—C19	−25.5 (3)
C7—C8—C9—O1	4.3 (3)	C17—C18—C19—C20	4.3 (3)
O1—C9—C10—C15	−13.8 (3)	C18—C19—C20—N1	18.3 (3)
O1—C9—C10—C11	167.5 (2)		

### Hydrogen-bond geometry (Å, º)

Cg3 is the centroid of the C10–C15 ring.

**Table:**

*D*—H···*A*	*D*—H	H···*A*	*D*···*A*	*D*—H···*A*
O2—H1*O*···N1	0.85 (3)	1.85 (3)	2.633 (2)	154 (3)
C5—H5···*Cg*3^i^	0.93	2.99	3.685 (2)	132

Symmetry code: (i) *x*+1/2, −*y*+3/2, −*z*.
